# Transfer Function between EEG and BOLD Signals of Epileptic Activity

**DOI:** 10.3389/fneur.2013.00001

**Published:** 2013-01-25

**Authors:** Marco Leite, Alberto Leal, Patrícia Figueiredo

**Affiliations:** ^1^Department of Bioengineering, Instituto Superior Técnico, Technical University of LisbonLisbon, Portugal; ^2^Institute for Systems and RoboticsLisbon, Portugal; ^3^Centro de Investigação e Intervenção SocialLisbon, Portugal; ^4^Department of Neurophysiology, Centro Hospitalar Psiquiátrico de LisboaLisbon, Portugal

**Keywords:** BOLD, EEG-fMRI, epilepsy, ICA, heuristic

## Abstract

Simultaneous electroencephalogram (EEG)-functional Magnetic Resonance Imaging (fMRI) recordings have seen growing application in the evaluation of epilepsy, namely in the characterization of brain networks related to epileptic activity. In EEG-correlated fMRI studies, epileptic events are usually described as boxcar signals based on the timing information retrieved from the EEG, and subsequently convolved with a hemodynamic response function to model the associated Blood Oxygen Level Dependent (BOLD) changes. Although more flexible approaches may allow a higher degree of complexity for the hemodynamics, the issue of how to model these dynamics based on the EEG remains an open question. In this work, a new methodology for the integration of simultaneous EEG-fMRI data in epilepsy is proposed, which incorporates a transfer function from the EEG to the BOLD signal. Independent component analysis of the EEG is performed, and a number of metrics expressing different models of the EEG-BOLD transfer function are extracted from the resulting time courses. These metrics are then used to predict the fMRI data and to identify brain areas associated with the EEG epileptic activity. The methodology was tested on both ictal and interictal EEG-fMRI recordings from one patient with a hypothalamic hamartoma. When compared to the conventional analysis approach, plausible, consistent, and more significant activations were obtained. Importantly, frequency-weighted EEG metrics yielded superior results than those weighted solely on the EEG power, which comes in agreement with previous literature. Reproducibility, specificity, and sensitivity should be addressed in an extended group of patients in order to further validate the proposed methodology and generalize the presented proof of concept.

## Introduction

Over the years, the electroencephalogram (EEG) has been the tool of choice for the diagnosis and characterization of epilepsy. With the possibility to acquire the EEG simultaneously with functional Magnetic Resonance Imaging (fMRI), studies of Blood Oxygen Level Dependent (BOLD) signals correlated with epileptic activity proliferated (Ives et al., 1993; Hoffmann et al., 2000; Lemieux et al., 2001; Salek-Haddadi et al., 2006; LeVan and Gotman, 2009). Despite its potential for the localization of epileptogenic brain networks in patients with drug-resistant focal epilepsy undergoing evaluation for surgical treatment, simultaneous EEG-fMRI has yet to reach its full potential in clinical practice. One factor contributing to this state of affairs is the lack of sensitivity in the identification of hemodynamic changes associated with the EEG epileptiform discharges in a significant number of studies (Aghakhani et al., 2006; Salek-Haddadi et al., 2006; Gotman, 2008; Grouiller et al., 2011). Although technical difficulties related with data acquisition and artifact correction may in part explain such results, probably most important are the limitations related with the remaining conceptual and methodological challenges associated with the integration of the two types of signals.

Although a great amount of both experimental and theoretical work has been dedicated to the clarification of the relationship between neural activity and associated hemodynamic changes, neurovascular coupling mechanisms remain an active area of research (Rosa et al., 2010a). The most consensual evidence comes from the recording of electrical activity using micro-electrodes implanted in the cortex of (non-human) animals, simultaneously with fMRI, indicating that the BOLD signal reflects mostly slow, post-synaptic input activity measured by local field potentials (LFPs), rather than fast, spiking output activity measured by single/multi-unit activity (S/MUA; Logothetis et al., 2001). In humans, a growing number of simultaneous EEG-fMRI studies on healthy subjects as well as epilepsy patients have now been reported (Goldman et al., 2002; Laufs et al., 2003, 2006; Moosmann et al., 2003; de Munck et al., 2009), and biophysical models of the neurovascular coupling have been proposed (Riera et al., 2006, 2007). Overall, reports in the literature do not provide a clear picture of the link between EEG and BOLD signals. In particular, contradictory results have been presented regarding the dependency of BOLD changes on the EEG power and spectral profiles. These include, for example, positive and negative BOLD correlations with specific frequency band power changes in the human EEG (de Munck et al., 2009), BOLD decoupling from LFP power in mice (Ekstrom, 2010), and negative BOLD associated with large increases in LFP and MUA during seizures also in mice (Schridde et al., 2008). Rosa et al. (2010b) addressed this topic by comparing different models of the transfer function between EEG and BOLD signals, in the prediction of fMRI data, in a visual stimulation experiment with human healthy subjects. The models explored included the EEG total power (TP; Wan et al., 2006); linear combinations of the power from different frequency bands (Goense and Logothetis, 2008); and several variations of a heuristic model proposed by Kilner et al. (2005) in which BOLD changes are assumed to be proportional to the root mean square frequency (RMSF) of the EEG spectrum. The results obtained showed a clear superiority of the RMSF metrics in predicting the BOLD signal, when compared to power-weighted metrics.

In most epilepsy EEG-fMRI studies, the goal is to find brain networks exhibiting hemodynamic changes associated with interictal and/or ictal activity, which are expected to be correlated with the epileptogenic areas (Hoffmann et al., 2000; Salek-Haddadi et al., 2006; Marques et al., 2009). Both ictal and interictal events are identified on the EEG trace and are then used to define regressors of interest in a general linear model (GLM) analysis of the fMRI data. Interictal spikes are usually described as stick functions and ictal activity as boxcar signals between seizure onset and offset, eventually sub-divided into up to three phases: early ictal EEG, clinical onset, and late ictal EEG. Both types of events are then convolved with a model of the hemodynamic response function (HRF; Tyvaert et al., 2008; Moeller et al., 2010; Thornton et al., 2010). Independent component analysis (ICA) of the fMRI data has also been performed in order to identify interictal/ictal BOLD patterns without resorting to the EEG (LeVan et al., 2010; Thornton et al., 2010). When the EEG accurately reflected the seizure onset, the GLM approach yielded activations concordant with the ictal onset zone; otherwise ICA still gave valuable insight on the ictal hemodynamics. Importantly, the question of whether or not the neurovascular coupling is preserved from healthy to disease conditions has also been investigated, by allowing variations in the HRF (Grouiller et al., 2010). It is in principle possible to achieve a higher degree of complexity for the epileptic activity hemodynamics by using more flexible HRFs, or completely model-free approaches such as ICA. However, such approaches do not address the issue of how to model these dynamics based on the information available in the EEG data (Lemieux, 2008).

In this paper, we address the issue of modeling the BOLD dynamics associated with epileptic EEG activity by extracting metrics from the EEG spectrum expressing different models of the transfer function between neuronal and hemodynamic signals. A methodology is proposed for the analysis of EEG-fMRI data in epilepsy, consisting of ICA decomposition of the EEG followed by component selection based the reproducibility across different acquisition runs, Morlet wavelet spectral analysis, and EEG metric extraction. The resulting time courses are convolved with a canonical HRF and used as regressors of interest in a GLM analysis of the fMRI data. The proposed methodology is applied to the study of a patient with epilepsy associated with a hypothalamic hamartoma. The different EEG-fMRI transfer functions are compared with each other, as well as with a conventional GLM methodology based on the identification of ictal and interictal events on the EEG by the neurophysiologist, and also with an fMRI-ICA approach.

## Materials and Methods

### Patient characterization

We focused on the simultaneous EEG-fMRI data recorded from a 2-year-old patient with a giant hypothalamic hamartoma suffering from gelastic epilepsy, as part of the pre-surgical evaluation under the Program for Epilepsy Surgery of the Hospital Center of West Lisbon. This case study has been previously described (Leal et al., 2009). This patient was studied in a run of 30 patients, from which only five had ictal events during the scanning sessions. From these five, only this one patient had an EEG trace clear from movement related MR artifacts triggered by the beginning of the seizures and therefore presented sufficient data quality for subsequent EEG quantification and was selected for this study.

Seizures occurred more than 50 times per day and typically lasted for 20–30 s, involving almost exclusively the left hemisphere. The clinical manifestations of the seizures consisted of slowing of motor activity, variable interruption of consciousness, eyelid rhythmic movements with bilateral nystagmus to the right, and occasionally gelastic laughter. After the acquisition of the EEG-fMRI data, the patient underwent a two-stage hamartoma disconnection surgery, 1 year after which the seizures were reduced to 1–3 per day.

The EEG interictal activity demonstrated a persistent slow-wave abnormality over the left temporal-occipital area, associated with abundant spike activity with occasional contralateral propagation. Abundant spike activity also occurred over the left hemisphere frontal lobe. Topological analysis of the interictal spikes presented a spatial stationarity for the posterior spikes, whereas the frontal ones changed significantly in configuration from an occipital dipolar potential at spike onset to a dipolar frontal potential at spike peak. The ictal EEG pattern was very monotonous and consisted of early diffuse desynchronization, followed by the build-up of spike activity over the left occipital and temporal areas and, in the later stages of the seizure, over the frontal area. Occasionally secondary propagation of spike activity to the right temporal areas occurred.

### Simultaneous EEG-fMRI acquisition

The EEG was recorded using an MR-compatible 37-channel system (Maglink, Neuroscan, Charlotte, NC, USA) with two ECG channels, using a sampling rate of 1000 Hz with a bandwidth DC-250 Hz and reference at electrode FCz. The imaging was performed on a 1.5 T MRI scanner (GE Cvi/NVi). Six fMRI runs were collected using a gradient-echo echoplanar imaging (EPI) sequence, with TR = 2.275 s, 3.75 mm × 3.75 mm × 5.00 mm voxel size and a total of 154 volumes (the first four were subsequently rejected in order to discard T_1_ relaxation unstationarities). A T_1_-weighted structural image was also acquired using a 3D spoiled gradient recovery (SPGR) sequence, with 0.94 mm × 0.94 mm in-plane resolution and 0.6 mm slice thickness. During the scanning session, the patient was administered light anesthesia with Sevoflurane at 1% (Abbott Laboratories, Abbot Park, IL, USA), through mask, as established by the MRI protocol for small children and uncooperative patients. A 5 min EEG recording was also performed outside the scanner and before anesthesia, in order to allow for cross-validation of the simultaneous EEG-fMRI recordings in terms of the presence of MR related artifacts and the effects of anesthesia. Periods of interictal and ictal activity were identified on the artifact-corrected EEG by the neurophysiologist. For runs 2 and 3, two and five ictal events occurred, respectively. For the remainder runs, only interictal spikes were detected on the EEG. The visual inspection of the EEG inside and outside the scanner revealed a slight increase in both beta and theta background activity under anesthesia, but there was no significant change in the morphology of interictal spikes.

### EEG analysis

#### Pre-processing

The EEG pre-processing was executed using the EEGLAB toolbox (Delorme and Makeig, 2004). Firstly, the EEG traces were visually inspected for the presence and consequent rejection of bad channels, associated with poor contacts. A 2 Hz high-pass filter was then applied so as to remove baseline drifts from the signal. The times of occurrence of the gradient artifacts associated with the acquisition of each fMRI slice were automatically identified on the EEG signals, by software developed in-house. The fMRI gradient artifact correction algorithm, FMRIB’s FASTR (Niazy et al., 2005), was then applied using the default parameters. For the pulse artifact removal, FMRIB’s QRS complex identification algorithm (Niazy et al., 2005) was first applied to the ECG channels. An optimal basis set of three principal components was then employed for pulse artifact removal of the data, after low-pass filtering at 45 Hz and down-sampling to 100 Hz to improve manageability.

#### ICA decomposition

In an attempt to separate out the activity of interest in the EEG, the pre-processed data were decomposed by ICA using the *infomax* algorithm as implemented in EEGLAB (Delorme and Makeig, 2004). The reference channel was arbitrarily kept as the one chosen by the electrophysiologist during the acquisition. Although the referencing method for the EEG channels does not affect the final IC time courses, because the reference channel is linearly separable from the data, it will affect the IC’s scalp topographies. Nevertheless, the reference channel was kept the same (FCz) throughout the acquisition of all six runs, so comparisons between the scalp topographies of components obtained in different runs are still possible.

A reproducibility analysis of the ICs was performed in order to identify the associated topographies that were consistent across the six acquisition runs. *IC groups* were built by fixing each IC of each run and selecting, for each of the other runs, the IC that was the most spatially correlated with it. A total of 6 runs × 25 ICs = 150 IC groups were hence generated, each composed of a string of six ICs. An IC group was considered to be *consistent* if the same associated string was generated by one IC of each run, and therefore was repeated six times in the total set of 150 IC groups. The topographies and time courses of the consistent IC groups were then visually inspected for the identification and consequent rejection of artifact related ICs, dominated by residual gradient artifacts, bad channels, or eye blink/movement artifacts.

#### EEG metrics

The spectral profiles of the selected ICs were obtained by time-frequency analysis through convolution of the respective time courses with Morlet wavelets. The subset of metrics found to be more relevant in Rosa et al. (2011) were applied here: mean frequency (MF), RMSF, un-normalized mean frequency (uMF) un-normalized mean square frequency (uRMSF), and TP.

The difference between frequency averaging measures (RMSF or MF) was found to have a negligible effect on BOLD signal prediction; hence the results emerging from the MF and uMF metrics will be omitted, as they were not significantly different from those of the RMSF and uRMSF metrics, respectively.

### fMRI analysis

The fMRI data were analyzed using FSL^1^, including: (1) pre-processing; (2) GLM; and (3) ICA.

Pre-processing consisted on: motion correction using MCFLIRT (Jenkinson et al., 2002); slice timing correction using (Hanning-windowed) sinc interpolation to shift each time-series by an appropriate fraction of a TR relative to the middle of the TR period; brain extraction using BET (Smith, 2002); temporal high-pass filtering rejecting periods above 100 s; and spatial Gaussian filtering with FWHM = 8 mm.

Two GLM analyses were specified in order to identify BOLD signal changes associated with: (1) the *EEG metrics* for each consistent IC group (as defined in the previous section); and (2) the epileptic activity identified by the neurophysiologist on the pre-processed EEG traces, where boxcar signals were used to describe the periods of ictal activity and stick functions were used to describe interictal spikes (from now on referred to as *EA*). Each of these variables of interest (*EEG metrics* and *EA*) was convolved with a canonical HRF (Friston et al., 1998), and the time and dispersion derivatives were also included to account for some degree of variability in the HRF shape across the patient’s brain. This resulted in a set of three regressors for each variable. The final GLM regressors were obtained by re-sampling the resulting time courses to match the middle of the acquisition time period of each fMRI volume. Six motion parameters were also included as regressors of no interest, in order to account for residual motion-related signal jitter not removed by the motion correction procedure. The GLM’s were fitted to the data using the FILM algorithm (Woolrich et al., 2001) *F* tests were then applied to each estimated parameter contrast, resulting in *Z* (Gaussianized *F*) statistic maps. These were thresholded using a clustering procedure, whereby each cluster is determined by a voxel *Z* > 2.3 and a (corrected) cluster significance threshold *p* = 0.05.

An ICA of each fMRI run was also performed using Probabilistic ICA as implemented in MELODIC (Multivariate Exploratory Linear Decomposition into Independent Components) Version 3.09, part of FSL (FMRIB’s Software Library (see text footnote 1); Beckmann and Smith, 2004). Pre-processing included voxel-wise de-meaning of the data and normalization of the voxel-wise variance. Estimated component maps were divided by the standard deviation of the residual noise and thresholded by fitting a mixture model to the histogram of intensity values.

### Model comparisons

The comparison between EEG metrics was performed through GLM analysis followed by inferences based on *F* tests, for each consistent IC group of interest and for each acquisition run. We first considered a single GLM comprising the three EEG metrics (TP, RMSF, and uRMSF) with three regressors of interest each (canonical HRF, time, and dispersion derivatives), totaling nine regressors of interest, in one three-way comparison. Three GLM’s contrasting pairs of EEG metrics (TP vs. RMSF, TP vs. uRMSF, and RMSF vs. uRMSF) were also considered, totaling six regressors of interest each, in three two-way comparisons. *F* tests were computed for each set of three regressors regarding each metric, as well as for the whole set of metrics. The resulting *Z* statistical maps were inspected for their volume, i.e., the total number of voxels exhibiting significant EEG metric-related BOLD changes. This was used as a quantitative measure of the performance of each EEG metric in terms of BOLD prediction, and the analysis was repeated for each consistent IC group.

The comparison between consistent IC groups of interest was performed in a similar way, for each acquisition run, applying the selected EEG metric. Here, we considered a single GLM comprising the three consistent IC groups of interest (I, II, and V, as will be shown in the Results) with three regressors of interest each (canonical HRF, time, and dispersion derivatives), totaling nine regressors of interest, in one three-way comparison.

In order to assess the plausibility and consistency of the EEG metric-derived BOLD maps, the EEG metric/IC group combination yielding the largest maps (and hence best at predicting BOLD signal changes) was selected for subsequent comparison with the GLM analysis using the EEG EA regressors and also with the ICA analyses. The consistency between each two regressors was measured as their temporal correlation. The consistency between two maps was measured as their spatial overlap, i.e., the ratio of the volume of the map intersection with the volume of the map union.

## Results

In this section, the results obtained through the proposed methodology will be presented.

### EEG analysis

**Figure 1:**
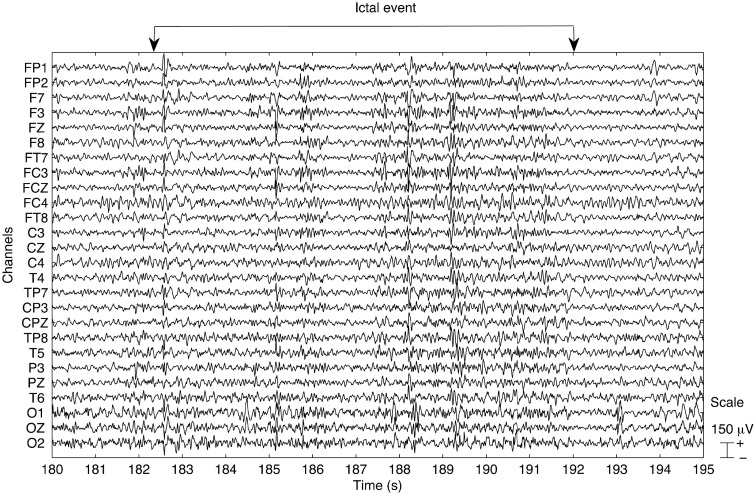
**Example of the EEG trace obtained after pre-processing the data for run 3**. An ictal event occurs within the time window starting at 182 s and ending at 193 s, as indicated. The data appear to be clear of MR related artifacts.

An exerpt of the EEG trace obtained after pre-processing is presented in Figure 1. The data appears to be clear from MR related artifacts and the ictal activity can be clearly identified.

**Figure 2:**
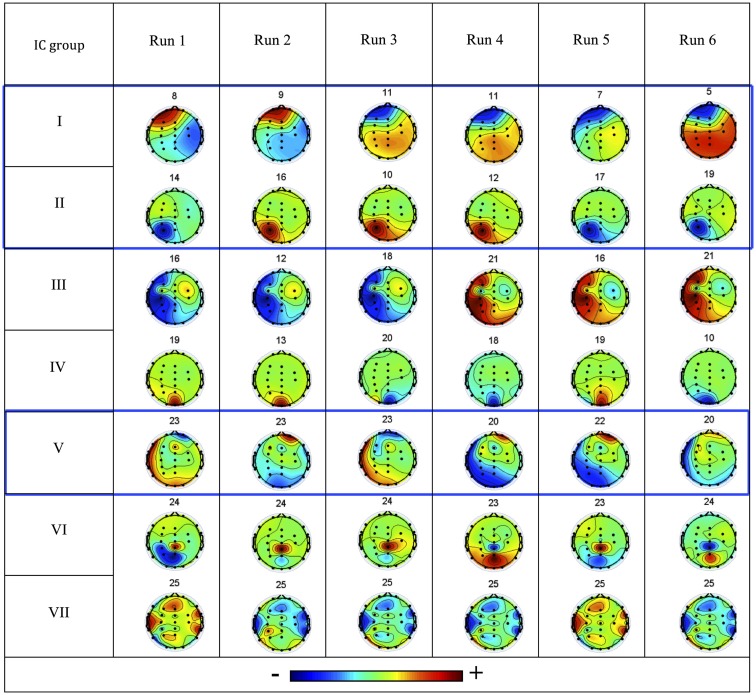
**Consistent IC groups I–VII: the associated IC topographies for each run are shown (color scale indicates relative weight of each EEG channel), together with the respective IC number**. The three IC groups highlighted in blue boxes were considered not artifactual and hence of interest (left is left, right is right, up is anterior, down is posterior).

The consistent IC groups, obtained as a function of the reproducibility of the associated IC topographies across runs, are presented in Figure 2. The IC groups I, II, and V were considered artifact free and were kept for further analysis, while the remainder were rejected. The IC groups I and II exhibit clear dipolar configurations in the left hemisphere, compatible with the frontal and occipital/parietal patterns of the patient’s interictal and ictal EEG. Given its predominantly frontal topography, particular attention was given to IC group II in order to confirm that ocular movement artifacts did not dominate the IC time course. Topography group V exhibits a more diffuse configuration difficult to interpret.

**Figure 3:**
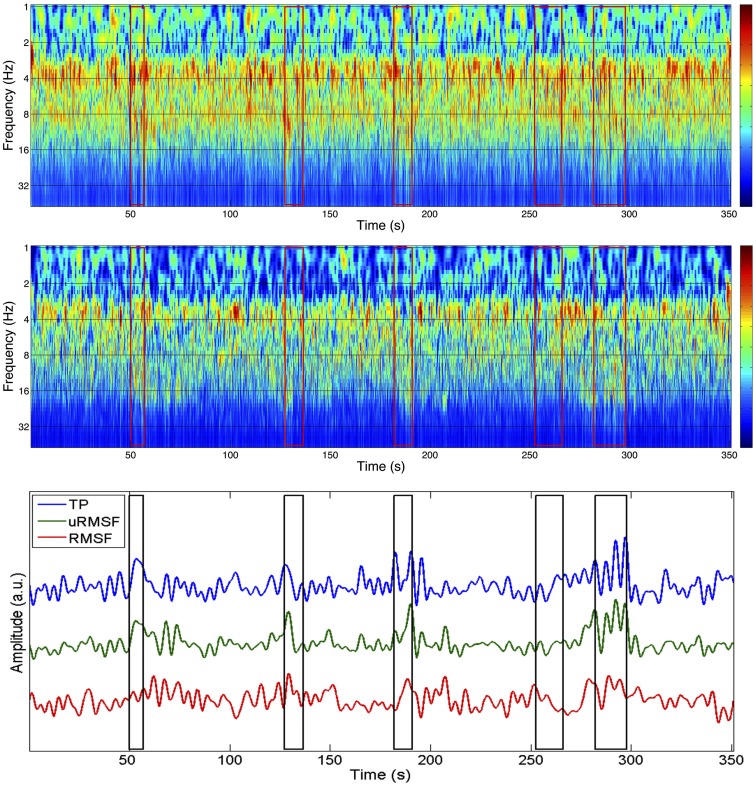
**Spectrograms of the time courses of channel P3 (top) and IC 10 (group II; middle) with corresponding EEG metrics (bottom), for the EEG of run 3**. The P3 channel was the one that contributed the most to IC 10. The boxes in red/black represent the periods identified as ictal events.

The spectral profiles obtained with Morlet wavelet decomposition for one IC (IC10) in run 3, as well as the EEG channel with highest absolute weight for this component (P3), are shown in Figure 3. Spectral changes associated with the ictal events identified by the neurophysiologist are visible in both spectrograms; however, these changes are clearer in the spectrogram obtained for the IC in comparison to the spectrogram of channel P3, or any other single EEG channel (data not shown). This observation suggests that ICA decomposition is capable of separating out the ictal activity spread over several EEG channels into a limited number of components.

### fMRI analysis

The BOLD statistical maps obtained using each EEG metric and consistent IC group were generally consistent with each other, across runs and also with the patient’s seizure semiology, but differed considerably in terms of the number of voxels showing statistically significant EEG metric-related BOLD changes. Firstly, the results of the comparison between the fMRI analysis using the different EEG metrics and consistent IC groups will be presented. A single metric and IC group will then be selected for comparison with the EA GLM and fMRI-ICA analyses.

#### EEG metric comparisons

**Figure 4:**
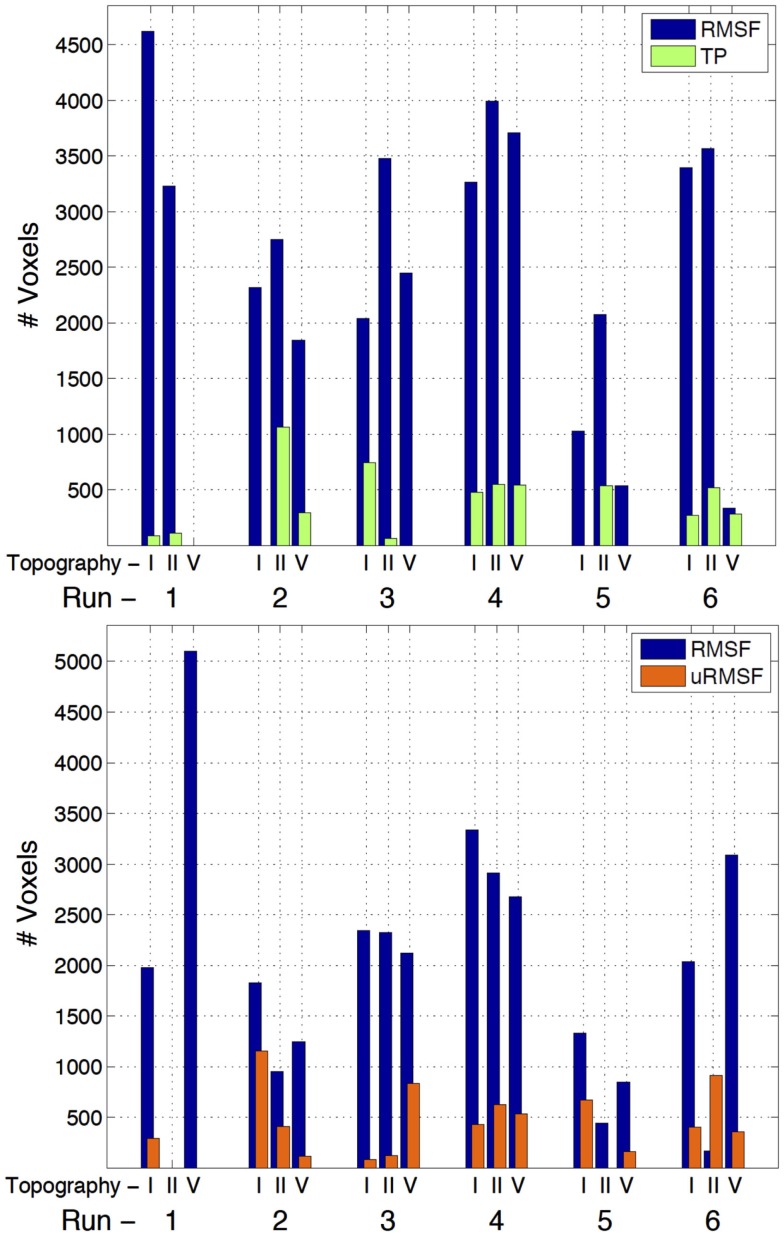
**Number of voxels exhibiting statistically significant EEG metric-related BOLD changes, for the two-way comparisons RMSF vs. TP (top) and RMSF vs. uRMSF (bottom), for each IC group (II, V, and I) and each run (1–6)**.

The three-way analysis did not show meaningful activations to allow for the comparison of the individual EEG metrics; however, the two-way comparisons yielded a clear superiority of the RMSF metric when compared to the TP and uRMSF metrics, for every IC group and run, in terms of the number of voxels showing statistically significant EEG metric-related BOLD changes, as shown in Figure 4.

**Figure 5:**
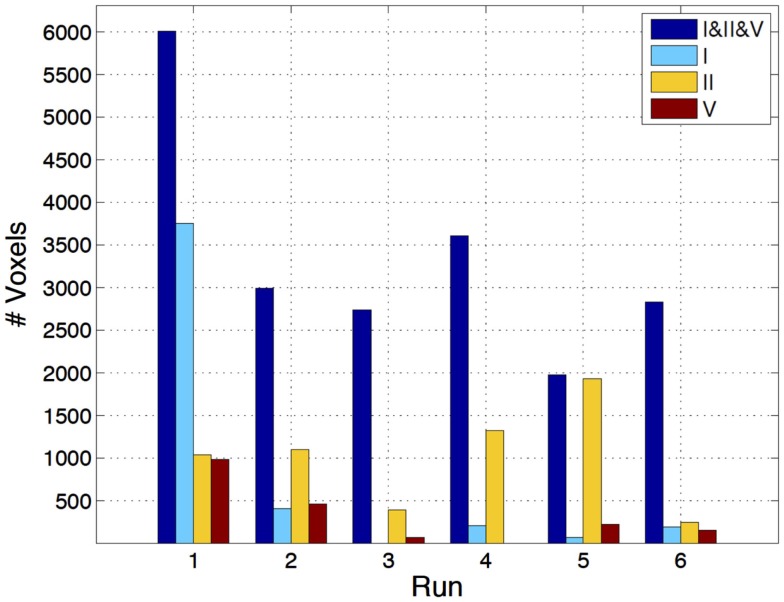
**Number of voxels exhibiting statistically significant RMSF-related BOLD changes, for the three-way analysis of IC groups I, II, and V, for each run (1–6)**.

The three-way comparison regarding the IC group showed a significant superiority of IC group II, in terms of the number of voxels exhibiting statistically significant EEG metric-related BOLD changes, as shown in Figure 5. For five out of the six runs, the statistical maps yielded by IC group II had larger volumes of significant voxels than those yielded by other ICs. The EEG RMSF metric of IC group II was found to be the best at predicting BOLD changes and it will now be compared with the results of the EA GLM analysis as well as with those of the fMRI-ICA approach.

**Figure 6:**
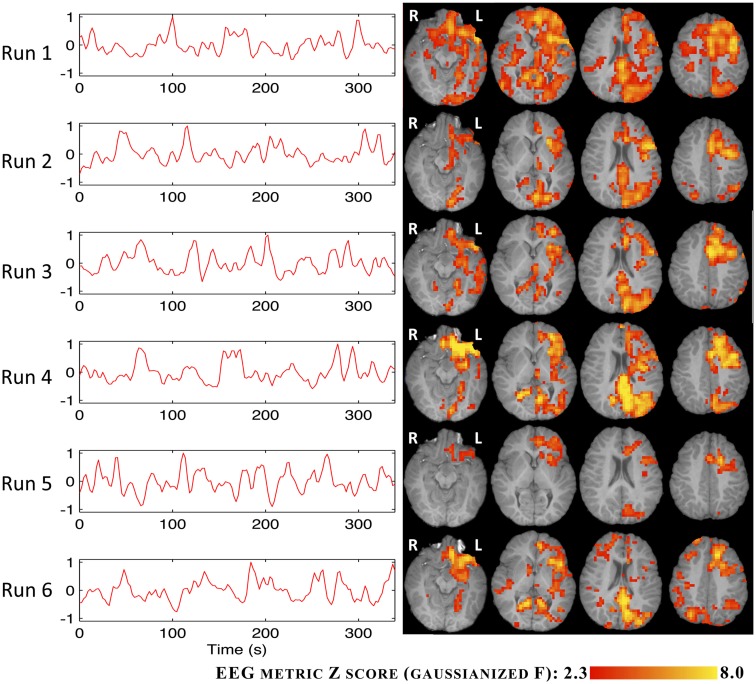
**Time courses (left) and *Z* statistic maps (right) of the RMSF metric for IC group I**.

**Figure 7:**
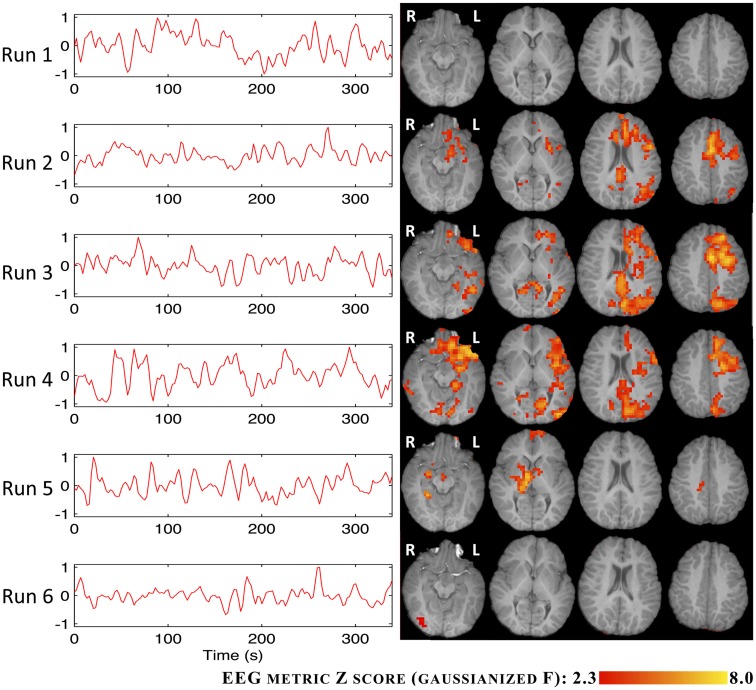
**Time courses (left) and *Z* statistic maps (right) of the RMSF metric for IC group V**.

The BOLD statistical maps obtained using the RMSF metric for IC groups I and V are shown in Figures 6 and 7.

#### EEG metric vs. EA GLM analysis

**Figure 8:**
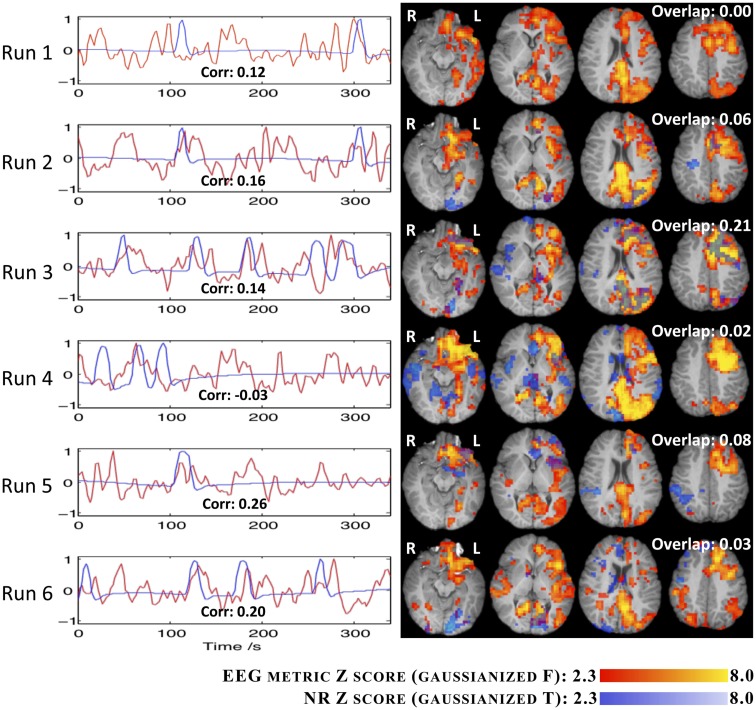
**Time courses (left) and *Z* statistic maps (right) of the EA (blue) and the RMSF metric for IC group II (red)**. The correlation between the two regressors (bottom of graphs) and the map overlap (top right of maps) are shown.

The comparison between the fMRI results obtained using the EEG RMSF metric and the EA GLM analysis, for IC group II, are summarized in Figure 8. For each run, the regressors associated with the canonical HRF are plotted alongside with the *Z* (Gaussianized *F*) statistic maps obtained with both methods. The temporal correlation between the regressors and the spatial overlap between the respective maps are also presented. The consistency between the two methodologies is generally low for all runs (correlation < 0.3 and overlap < 3), with only run 3 exhibiting an overlap between maps above 10%. Interestingly, this is also the run during which more ictal events occurred.

In general, the maps obtained using the EEG metric approach were consistent across runs and also with the patient’s seizure semiology. In fact, clusters were found on the left occipital/parietal and left frontal lobes, consistently with the observation on the EEG of spike buildups in left occipital/parietal channels followed by the left frontal channels, after a generalized desynchronization. In run 3, the EA approach was also capable of identifying these brain areas, but the corresponding statistical maps were more significant and extensive for the EEG metric approach. Furthermore, with the EEG metric approach, clusters involving left thalamic, left hippocampal, and left frontal ventral areas, as well as the hamartoma itself, were also found, which are generally consistent with the brain network associated with seizures originating in a hypothalamic hamartoma. For the remaining runs, in contrast with the EEG metric approach, the fMRI statistical maps obtained with the EA approach were in general not consistent across runs nor with the expected epileptic network.

#### EEG metric vs. fMRI-ICA approach

**Figure 9:**
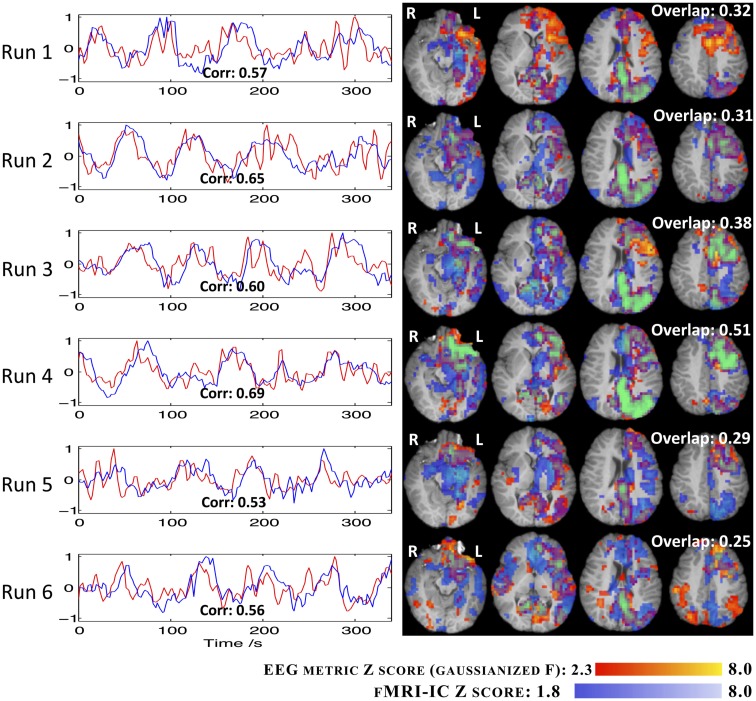
**Time courses (left) and *Z* statistic maps (right) of the fMRI-IC (blue) and the RMSF metric for IC group II (red)**. The correlation between the two regressors (bottom of graphs) and the map overlap (top right of maps) are shown.

The results regarding the EEG metric GLM analysis and the fMRI-ICA decomposition of all the runs, for IC group II, are presented in Figure 9. The fMRI-ICs presented are those that yielded the highest spatial map overlap with the results of the corresponding EEG metric analysis. Map spatial overlaps and time course temporal correlations between EEG metric GLM and fMRI-ICA approaches were generally higher than those found between EEG metric GLM and EA GLM approaches (Figure 8). Moreover, in contrast with the EA approach, with fMRI-ICA, consistency across runs was also observed for the brain areas expected in the patient’s epileptic network. Interestingly, runs where only interictal activity was recorded (1, 4, 5, and 6) yielded maps consistent with runs with ictal activity (2 and 3).

## Discussion

We proposed a new methodology for BOLD signal prediction in EEG-correlated fMRI studies in epilepsy, by incorporating a model of the EEG-BOLD transfer function. Specifically, independent components of the EEG associated with consistent topographies were translated into BOLD signal predictions by a set of model-based metrics. Interestingly, we found that increases in the MF of the EEG were better than its power at predicting BOLD increases, in support of the heuristic proposed in (Kilner et al., 2005) and in agreement with the results obtained in a visual stimulation experiment with healthy subjects (Rosa et al., 2010b). Moreover, such EEG-based metrics were found to improve detection sensitivity compared with conventional approaches to EEG-fMRI data analysis in epilepsy.

The heuristic proposed by Kilner and colleagues puts forward the MF (in an RMS sense) of the EEG spectrum as a surrogate of the neuronal activity eliciting the BOLD signal, following a broad physiological inspiration: the BOLD eliciting signal is assumed to be proportional to the electric work dissipated by the ionic currents across the cell membranes; this can in turn be shown to be proportional to the RMSF of the LFP/EEG spectrum if the covariance of the membrane potentials of the cells are assumed constant (Kilner et al., 2005). Although more detailed models of EEG-fMRI integration have been presented in the literature (Riera et al., 2006, 2007), the heuristic benefits from its simplicity in application and interpretability. The dependency of the BOLD signal on the EEG spectral profile has often been experimentally reported in studies of spontaneous or evoked fluctuations of brain rhythms, and the results are most frequently found to be in concordance with Kilner’s heuristic predictions (Goldman et al., 2002; Gonçalves et al., 2006; Laufs et al., 2006; de Munck et al., 2009; Michels et al., 2010; Zumer et al., 2010; Khursheed et al., 2011). Rosa et al. (2010b) have explicitly employed the heuristic to analyze EEG-fMRI data collected during a visual flicker stimulation task in healthy subjects, and showed that BOLD signal decreases were indeed associated with changes in the EEG spectral profile, namely its RMSF, which did not arise from power changes in one specific frequency band. In epilepsy, low-frequency slow-wave activity increases have been shown to be associated with BOLD decreases (Archer et al., 2003) while high-frequency spike and wave discharges have been shown to be associated with BOLD increases (Krakow et al., 2001; Hamandi et al., 2004). Although these finding are in agreement with the heuristic, our study is the first one to test it explicitly on EEG-fMRI data of epileptic activity.

The EEG metric-related BOLD change maps were consistent with the ones obtained using the epileptic activity regressors defined by the neurophysiologist, whenever ictal activity was identified on the EEG. For the runs on which no ictal events were detected on the EEG, the proposed methodology was able to identify the same brain network that was involved in the ictal runs. This was achieved by regressors based on the same IC scalp topography as those from the ictal runs, suggesting the presence of underlying epileptic activity in the identified epileptic network, which only occasionally manifests itself with an ictal character. In contrast with the proposed EEG metric based approach, however, the conventional analysis of the interictal runs yielded inconsistent or no results for the same statistical significance threshold. These findings suggest that the interictal events detected on the EEG may not fully reflect the activity of the underlying brain network, while the selected EEG metrics may be more powerful in depicting it. The same network was also identified by fMRI-ICA on both ictal and interictal runs, which further supports this idea. However, the fully data-driven method of fMRI-ICA lacks an explanatory model for the data, in contrast with the proposed methodology, which is based on modeling the EEG-BOLD transfer function.

For the purpose of verifying the specific epileptic character of the identified brain networks, their consistency across runs and their plausibility as the epileptic brain network underlying the patient’s seizure semiology were considered. The overlap of the corresponding BOLD change maps across runs was very high, both for the EEG metrics GLM and the fMRI-ICA approaches. Moreover, these maps were in good agreement with the brain network known to be involved in the epileptic activity of this patient, which includes the hamartoma, as well as left hemisphere hypothalamus, hippocampus, parietal–occipital lobe, cingulate gyrus, and dorsal–lateral frontal lobe (Leal et al., 2009). Clusters in the left parietal–occipital and frontal lobes are consistent with the observation on the patient’s EEG of spike buildups in left parietal–occipital channels followed by the left frontal channels, after a generalized desynchronization. Clusters involving left thalamus and hippocampus, as well as the hamartoma itself, are generally consistent with the brain network associated with seizures originating in a hypothalamic hamartoma (Leal et al., 2003). Future work should be carried out in order to further validate the networks identified by our methodology. On a first approach, the topographies of the selected ICs could be compared with those obtained by ICA decomposition of the EEG performed outside the MRI scanner. Ultimately, intra-cranial EEG recordings (unavailable for this patient) must be used in order to achieve a conclusive validation.

An ICA decomposition of the EEG was used here with the purpose of separating out the activity of interest into a univariate timecourse, which was expected to exhibit a consistent and meaningful topography. ICA is a popular technique for the removal of muscular activity or eye movement artifacts in EEG data processing (Vigario, 1997). Because of the large amplitude of interictal epileptic activity and the fact that its sources can generally be assumed to be spatially stationary, ICA has also proved to be useful in the separation and identification of such activity (Kobayashi et al., 1999; Urrestarazu et al., 2006; Marques et al., 2009; Formaggio et al., 2011). However, ICA decomposition of EEG ictal events that involve spatial propagation may be questionable in the sense that the spatial stationarity assumption of the EEG sources is not verified. This is in fact the case in our study, where ICA was applied to the EEG recorded during seizures exhibiting a spatial propagation pattern associated (Leal et al., 2009). The results obtained may reflect this issue to some extent, as no single IC isolated *per se* all of the seizure dynamics. Nevertheless, ICA was useful for the separation of local approximately stationary activity, giving the ICs higher signal-to-noise ratio for the signals of interest, by separating them from activity in neighboring brain regions and also from residual artifacts not fully corrected by the EEG pre-processing as observable in the spectral analyses and scalp topographies.

Other approaches for the extraction of a univariate EEG time course, representative of the epileptic activity, have been proposed in the literature, namely continuous Electrical Source Imaging (Vulliemoz et al., 2010) and weighted averaging of selected ICs (Formaggio et al., 2011). The former corresponds to the projection of the recorded EEG data into the space of an EEG source estimation, which provides a more informed way of obtaining the EEG source signal than ICA. However, this technique is more prone to include artifacts in the resultant time courses when compared to ICA, as the latter automatically rejects channels contaminated with relevant artifacts. Regarding the averaging of selected EEG IC time courses, there is no straightforward way to compute averaging weights. Furthermore, when averaging ICs with discrepant topographies, one incurs in the risk of mixing sources with significantly different dynamics and loosing the meaning of the ICA source separation.

A limitation of the proposed approach is the bias of the EEG measures toward superficial cortical activity, which possibly precludes the identification of hemodynamic changes associated with deep brain activity. This limitation is however common to all EEG-fMRI “integration through prediction” approaches. Nonetheless, the particular syndrome of epilepsy associated with hypothalamic hamartomas is rather well described in the literature, with clear evidence for the hypothalamic hamartoma being the seizure focus, and this region was in fact found in our work in five out of six EEG-fMRI datasets. Although the proposed EEG-BOLD transfer functions are not specific to epileptic activity, this specificity is achieved in the presented methodology by selecting the EEG topography used to extract the metric based on an ICA procedure. Nevertheless, a possible limitation of the proposed transfer functions is that they are not specific to the start of the seizure, and hence to the epileptogenic focus. However, our aim was the description of the full epileptic network, leaving the specific identification of the seizure focus and seizure propagation dynamics for other, related lines of research (Murta et al., 2012).

The study presented here focused on data from a single patient with the aim of providing a proof of concept for the potential usefulness of the proposed methodology for the identification of epileptic networks. The choice of this patient was based on the quality of the EEG data that could be achieved due to the absence of movement associated with the beginning of the seizures. Moreover, epilepsy cases associated with hypothalamic hamartomas are relatively stereotypical in terms of the electrophysiological patterns of seizure propagation, which makes the interpretation of the results relatively more robust in comparison to other types of epilepsy. In general, the clinical utility of EEG-fMRI is yet to be established. Nevertheless, in this case study, the results of the EEG-fMRI investigation indicate possible alternative treatment approaches involving the surgical interruption of the seizure propagation pathways (Leal et al., 2009; Murta et al., 2012). The proposed methodology should now be applied to an extended group of patients, in order to generalize the proof of concept presented here and to further validate it. Reproducibility, specificity, and sensitivity should then also be addressed.

In conclusion, we presented a new approach for EEG-fMRI integration in the field of epilepsy, which incorporates and tests different models of the transfer function between EEG and BOLD signals, hence allowing better predictions of the hemodynamic changes associated with epileptic activity. This work therefore provides a contribution to our understanding of the link between EEG and BOLD signals as well as for improving the yield of EEG-fMRI studies in epilepsy.

## Conflict of Interest Statement

The authors declare that the research was conducted in the absence of any commercial or financial relationships that could be construed as a potential conflict of interest.

## Footnotes

^1^www.fmrib.ox.ac.uk/fsl

## References

[B1] Aghakhani Y. Kobayashi E. Bagshaw A. P. Hawco C. Benar C. G. Dubeau F. (2006). Cortical and thalamic fMRI responses in partial epilepsy with focal and bilateral synchronous spikes. Clin. Neurophysiol. 117, 177–191 10.1016/j.clinph.2005.08.028 16314143

[B2] Archer J. S. Abbott D. F. Waites A. B. Jackson G. D. (2003). fMRI “deactivation” of the posterior cingulate during generalized spike and wave. Neuroimage 20, 1915–1922 10.1016/S1053-8119(03)00294-5 14683697

[B3] Beckmann C. F. Smith S. M. (2004). Probabilistic independent component analysis for functional magnetic resonance imaging. IEEE Trans. Med. Imaging 23, 137–152 10.1109/TMI.2003.822821 14964560

[B4] de Munck J. C. Goncalves S. I. Mammoliti R. Heethaar R. M. Lopes Da Silva F. H. (2009). Interactions between different EEG frequency bands and their effect on alpha-fMRI correlations. Neuroimage 47, 69–76 10.1016/S1053-8119(09)70400-8 19376236

[B5] Delorme A. Makeig S. (2004). EEGLAB: an open source toolbox for analysis of single-trial EEG dynamics including independent component analysis. J. Neurosci. Methods 134, 9–21 10.1016/j.jneumeth.2003.10.009 15102499

[B6] Ekstrom A. (2010). How and when the fMRI BOLD signal relates to underlying neural activity: the danger in dissociation. Brain Res. Rev. 62, 233–244 10.1016/j.brainresrev.2009.12.004 20026191 PMC3546820

[B7] Formaggio E. Storti S. F. Bertoldo A. Manganotti P. Fiaschi A. Toffolo G. M. (2011). Integrating EEG and fMRI in epilepsy. Neuroimage 54, 2719–2731 10.1016/j.neuroimage.2010.11.038 21109007

[B8] Friston K. J. Fletcher P. Josephs O. Holmes A. Rugg M. D. Turner R. (1998). Event-related fMRI: characterizing differential responses. Neuroimage 7, 30–40 10.1006/nimg.1997.0306 9500830

[B9] Goense J. B. Logothetis N. K. (2008). Neurophysiology of the BOLD fMRI signal in awake monkeys. Curr. Biol. 18, 631–640 18439825 10.1016/j.cub.2008.03.054

[B10] Goldman R. I. Stern J. M. Engel J. Jr. Cohen M. S. (2002). Simultaneous EEG and fMRI of the alpha rhythm. Neuroreport 13, 2487–2492 12499854 10.1097/01.wnr.0000047685.08940.d0 PMC3351136

[B11] Gonçalves S. I. de Munck J. C. Pouwels P. J. Schoonhoven R. Kuijer J. P. Maurits N. M. (2006). Correlating the alpha rhythm to BOLD using simultaneous EEG/fMRI: inter-subject variability. Neuroimage 30, 203–213 16290018 10.1016/j.neuroimage.2005.09.062

[B12] Gotman J. (2008). Epileptic networks studied with EEG-fMRI. Epilepsia 49(Suppl. 3), 42–51 18304255 10.1111/j.1528-1167.2008.01509.x PMC3792078

[B13] Grouiller F. Thornton R. C. Groening K. Spinelli L. Duncan J. S. Schaller K. (2011). With or without spikes: localization of focal epileptic activity by simultaneous electroencephalography and functional magnetic resonance imaging. Brain 134, 2867–2886 21752790 10.1093/brain/awr156 PMC3656675

[B14] Grouiller F. Vercueil L. Krainik A. Segebarth C. Kahane P. David O. (2010). Characterization of the hemodynamic modes associated with interictal epileptic activity using a deformable model-based analysis of combined EEG and functional MRI recordings. Hum. Brain Mapp. 31, 1157–1173 20063350 10.1002/hbm.20925 PMC6871024

[B15] Hamandi K. Salek-Haddadi A. Fish D. R. Lemieux L. (2004). EEG/functional MRI in epilepsy: the queen square experience. J. Clin. Neurophysiol. 21, 241–248 10.1097/00004691-200407000-00002 15509913

[B16] Hoffmann A. Jager L. Werhahn K. J. Jaschke M. Noachtar S. Reiser M. (2000). Electroencephalography during functional echo-planar imaging: detection of epileptic spikes using post-processing methods. Magn. Reson. Med. 44, 791–798 10.1002/1522-2594(200011)44:5<791::AID-MRM17>3.0.CO;2-2 11064414

[B17] Ives J. R. Warach S. Schmitt F. Edelman R. R. Schomer D. L. (1993). Monitoring the patient’s EEG during echo planar MRI. Electroencephalogr. Clin. Neurophysiol. 87, 417–420 7508375 10.1016/0013-4694(93)90156-p

[B18] Jenkinson M. Bannister P. Brady M. Smith S. (2002). Improved optimization for the robust and accurate linear registration and motion correction of brain images. Neuroimage 17, 825–841 10.1006/nimg.2002.1132 12377157

[B19] Khursheed F. Tandon N. Tertel K. Pieters T. A. Disano M. A. Ellmore T. M. (2011). Frequency-specific electrocorticographic correlates of working memory delay period fMRI activity. Neuroimage 56, 1773–1782 21356314 10.1016/j.neuroimage.2011.02.062 PMC3085578

[B20] Kilner J. M. Mattout J. Henson R. Friston K. J. (2005). Hemodynamic correlates of EEG: a heuristic. Neuroimage 28, 280–286 16023377 10.1016/j.neuroimage.2005.06.008

[B21] Kobayashi K. James C. J. Nakahori T. Akiyama T. Gotman J. (1999). Isolation of epileptiform discharges from unaveraged EEG by independent component analysis. Clin. Neurophysiol. 110, 1755–1763 10574290 10.1016/s1388-2457(99)00134-0

[B22] Krakow K. Messina D. Lemieux L. Duncan J. S. Fish D. R. (2001). Functional MRI activation of individual interictal epileptiform spikes. Neuroimage 13, 502–505 11170815 10.1006/nimg.2000.0708

[B23] Laufs H. Holt J. L. Elfont R. Krams M. Paul J. S. Krakow K. (2006). Where the BOLD signal goes when alpha EEG leaves. Neuroimage 31, 1408–1418 10.1016/j.neuroimage.2006.02.002 16537111

[B24] Laufs H. Kleinschmidt A. Beyerle A. Eger E. Salek-Haddadi A. Preibisch C. (2003). EEG-correlated fMRI of human alpha activity. Neuroimage 19, 1463–1476 10.1016/S1053-8119(03)00286-6 12948703

[B25] Leal A. J. Monteiro J. P. Secca M. F. Jordao C. (2009). Functional brain mapping of ictal activity in gelastic epilepsy associated with hypothalamic hamartoma: a case report. Epilepsia 50, 1624–1631 10.1111/j.1528-1167.2008.01810.x 19183218

[B26] Leal A. J. Moreira A. Robalo C. Ribeiro C. (2003). Different electroclinical manifestations of the epilepsy associated with hamartomas connecting to the middle or posterior hypothalamus. Epilepsia 44, 1191–1195 10.1046/j.1528-1157.2003.66902.x 12919391

[B27] Lemieux L. (2008). Causes, relationships and explanations: the power and limitations of observational longitudinal imaging studies. Curr. Opin. Neurol. 21, 391–392 10.1097/WCO.0b013e3283056a50 18607197

[B28] Lemieux L. Salek-Haddadi A. Josephs O. Allen P. Toms N. Scott C. (2001). Event-related fMRI with simultaneous and continuous EEG: description of the method and initial case report. Neuroimage 14, 780–787 10.1006/nimg.2001.0896 11506550

[B29] LeVan P. Gotman J. (2009). Independent component analysis as a model-free approach for the detection of BOLD changes related to epileptic spikes: a simulation study. Hum. Brain Mapp. 30, 2021–2031 10.1002/hbm.20647 18726909 PMC3792083

[B30] LeVan P. Tyvaert L. Moeller F. Gotman J. (2010). Independent component analysis reveals dynamic ictal BOLD responses in EEG-fMRI data from focal epilepsy patients. Neuroimage 49, 366–378 10.1016/j.neuroimage.2009.07.064 19647798 PMC3779215

[B31] Logothetis N. K. Pauls J. Augath M. Trinath T. Oeltermann A. (2001). Neurophysiological investigation of the basis of the fMRI signal. Nature 412, 150–157 11449264 10.1038/35084005

[B32] Marques J. P. Rebola J. Figueiredo P. Pinto A. Sales F. Castelo-Branco M. (2009). ICA decomposition of EEG signal for fMRI processing in epilepsy. Hum. Brain Mapp. 30, 2986–2996 19172633 10.1002/hbm.20723 PMC6870975

[B33] Michels L. Bucher K. Lüchinger R. Klaver P. Martin E. Jeanmonod D. Brandeis D. (2010). Simultaneous EEG-fMRI during a working memory task: modulations in low and high frequency bands. PLoS One 5:e10298 10.1371/journal.pone.0010298 20421978 PMC2858659

[B34] Moeller F. Levan P. Muhle H. Stephani U. Dubeau F. Siniatchkin M. (2010). Absence seizures: individual patterns revealed by EEG-fMRI. Epilepsia 51, 2000–2010 10.1111/j.1528-1167.2010.02698.x 20726875 PMC3769289

[B35] Moosmann M. Ritter P. Krastel I. Brink A. Thees S. Blankenburg F. (2003). Correlates of alpha rhythm in functional magnetic resonance imaging and near infrared spectroscopy. Neuroimage 20, 145–158 10.1016/S1053-8119(03)00344-6 14527577

[B36] Murta T. Leal A. Garrido M. I. Figueiredo P. (2012). Dynamic causal modelling of epileptic seizure propagation pathways: a combined EEG-fMRI study. Neuroimage 62, 1634–1642 22634857 10.1016/j.neuroimage.2012.05.053 PMC3778869

[B37] Niazy R. K. Beckmann C. F. Iannetti G. D. Brady J. M. Smith S. M. (2005). Removal of FMRI environment artifacts from EEG data using optimal basis sets. Neuroimage 28, 720–737 16150610 10.1016/j.neuroimage.2005.06.067

[B38] Riera J. J. Jimenez J. C. Wan X. Kawashima R. Ozaki T. (2007). Nonlinear local electrovascular coupling. II: from data to neuronal masses. Hum. Brain Mapp. 28, 335–354 10.1002/hbm.20278 16933303 PMC6871399

[B39] Riera J. J. Wan X. Jimenez J. C. Kawashima R. (2006). Nonlinear local electrovascular coupling. I: a theoretical model. Hum. Brain Mapp. 27, 896–914 16729288 10.1002/hbm.20230 PMC6871312

[B40] Rosa M. J. Daunizeau J. Friston K. J. (2010a). EEG-fMRI integration: a critical review of biophysical modeling and data analysis approaches. J. Integr. Neurosci. 9, 453–476 10.1142/S0219635210002512 21213414

[B41] Rosa M. J. Kilner J. Blankenburg F. Josephs O. Penny W. (2010b). Estimating the transfer function from neuronal activity to BOLD using simultaneous EEG-fMRI. Neuroimage 49, 1496–1509 19778619 10.1016/j.neuroimage.2009.09.011 PMC2793371

[B42] Rosa M. J. Kilner J. M. Penny W. D. (2011). Bayesian comparison of neurovascular coupling models using EEG-fMRI. PLoS Comput. Biol. 7:e1002070 10.1371/journal.pcbi.1002070 21698175 PMC3116890

[B43] Salek-Haddadi A. Diehl B. Hamandi K. Merschhemke M. Liston A. Friston K. (2006). Hemodynamic correlates of epileptiform discharges: an EEG-fMRI study of 63 patients with focal epilepsy. Brain Res. 1088, 148–166 10.1016/j.brainres.2006.02.098 16678803

[B44] Schridde U. Khubchandani M. Motelow J. E. Sanganahalli B. G. Hyder F. Blumenfeld H. (2008). Negative BOLD with large increases in neuronal activity. Cereb. Cortex 18, 1814–1827 10.1093/cercor/bhm208 18063563 PMC2790390

[B45] Smith S. M. (2002). Fast robust automated brain extraction. Hum. Brain Mapp. 17, 143–155 10.1002/hbm.10062 12391568 PMC6871816

[B46] Thornton R. C. Rodionov R. Laufs H. Vulliemoz S. Vaudano A. Carmichael D. (2010). Imaging haemodynamic changes related to seizures: comparison of EEG-based general linear model, independent component analysis of fMRI and intracranial EEG. Neuroimage 53, 196–205 10.1016/j.neuroimage.2010.05.064 20570736

[B47] Tyvaert L. Hawco C. Kobayashi E. Levan P. Dubeau F. Gotman J. (2008). Different structures involved during ictal and interictal epileptic activity in malformations of cortical development: an EEG-fMRI study. Brain 131, 2042–2060 10.1093/brain/awn145 18669486 PMC3792088

[B48] Urrestarazu E. Iriarte J. Artieda J. Alegre M. Valencia M. Viteri C. (2006). Independent component analysis separates spikes of different origin in the EEG. J. Clin. Neurophysiol. 23, 72–78 16514354 10.1097/01.wnp.0000185243.35669.51

[B49] Vigario R. N. (1997). Extraction of ocular artefacts from EEG using independent component analysis. Electroencephalogr. Clin. Neurophysiol. 103, 395–404 10.1016/S0013-4694(97)00042-8 9305288

[B50] Vulliemoz S. Rodionov R. Carmichael D. W. Thornton R. Guye M. Lhatoo S. D. (2010). Continuous EEG source imaging enhances analysis of EEG-fMRI in focal epilepsy. Neuroimage 49, 3219–3229 19948231 10.1016/j.neuroimage.2009.11.055

[B51] Wan X. Riera J. Iwata K. Takahashi M. Wakabayashi T. Kawashima R. (2006). The neural basis of the hemodynamic response nonlinearity in human primary visual cortex: implications for neurovascular coupling mechanism. Neuroimage 32, 616–625 16697664 10.1016/j.neuroimage.2006.03.040

[B52] Woolrich M. W. Ripley B. D. Brady M. Smith S. M. (2001). Temporal autocorrelation in univariate linear modeling of FMRI data. Neuroimage 14, 1370–1386 10.1006/nimg.2001.0931 11707093

[B53] Zumer J. M. Brookes M. J. Stevenson C. M. Francis S. T. Morris P. G. (2010). Relating BOLD fMRI and neural oscillations through convolution and optimal linear weighting. Neuroimage 49, 1479–1489 10.1016/j.neuroimage.2009.09.020 19778617

